# How identity exploration may support freshman adaptation: a longitudinal within-person mediation through psychological resilience

**DOI:** 10.3389/fpsyg.2026.1841811

**Published:** 2026-07-08

**Authors:** Jiwen Chen, Yanting Huang

**Affiliations:** 1 Department of Counseling Psychology, General Graduate School, Dongshin University, Naju-si, Republic of Korea; 2 School of Asian and European Languages, Hainan College of Foreign Studies, Wenchang, Hainan, China

**Keywords:** college adaptation, college freshmen, emerging adulthood, psychological resilience, self-identity exploration

## Abstract

While constructive identity exploration (i.e., active, goal-directed engagement with identity alternatives in breadth and depth) is widely recognized as a critical developmental task during the transition to college, the within-person temporal mechanisms through which it relates to adaptation outcomes remain poorly understood. This study addressed this gap by examining the longitudinal within-person associations among constructive self-identity exploration, psychological resilience, and college adaptation among emerging adults during the college transition. A three-wave longitudinal design with a sample of Chinese freshmen (*N* = 665, Mean age = 19.01, SD = 0.81) was utilized, employing a Random Intercept Cross-Lagged Panel Model to disentangle within-person processes from stable between-person differences. Results indicated that initial exploration did not directly predict subsequent college adaptation. Instead, a significant longitudinal indirect pathway emerged at the intra-individual level [est = 0.085, 95% CI (0.044, 0.135)]: constructive exploration positively predicted subsequent psychological resilience, which in turn was positively associated with better college adaptation. While college adaptation showed strong autoregressive stability reflecting the tendency for adjustment patterns to persist over time, psychological resilience also exhibited robust, self-sustaining carry-over effects. Furthermore, the predictive association of resilience with subsequent college adaptation strengthened significantly over time. Additionally, a reciprocal indirect pathway from initial adaptation to later resilience through exploration was also observed, suggesting a dynamic developmental process. These findings suggest that identity exploration may contribute to college adaptation not directly but through building accumulable psychological resources, offering preliminary insights potentially applicable to university support systems across diverse educational contexts.

## 1 Introduction

The college adaptation issues faced by first-year students are widespread in higher education systems around the world (Clinciu, 2013; Noman et al., 2023). Research consistently demonstrates that poor adaptation during the freshman year is associated with increased risk of academic underperformance, mental health difficulties, and dropout (Credé and Niehorster, 2012; Respondek et al., 2017). Understanding the developmental processes that contribute to successful adaptation is therefore of considerable theoretical and practical importance. In China, first-year students often experience significant difficulties in adapting to university life, characterized by academic pressure, social isolation, and emotional distress, which can lead to depression, anxiety, or dropping out (He and Xu, 2022; Ti et al., 2025). In developmental psychology, the transition to college aligns with the period of emerging adulthood (ages 18–25), which is recognized as a distinct developmental stage characterized by profound identity exploration, instability, and a focus on the self (Arnett, 2000, 2014). Through exploring values, goals, and possible life directions, young people gradually construct a coherent sense of self, which is often associated with autonomy, meaning, and well-being (Schwartz et al., 2023).

In the Chinese context, the educational trajectory creates unique developmental patterns driven by the National College Entrance Examination (Gaokao). During secondary education, Chinese students typically focus on academic performance to secure university admission, often suppressing personal interests in a highly structured environment (Liu and Helwig, 2020). Consequently, the transition to university marks an abrupt shift from external control to sudden autonomy, precipitating a period of delayed identity exploration (Liu, 2022). Chinese freshmen must navigate this exploration simultaneously with the demands of adaptation, making the integration of identity exploration into academic and social life complex (Qiu et al., 2025).

Existing research has primarily relied on cross-sectional designs to investigate the direct associations between identity exploration and adaptation outcomes (Oh and Jeong, 2021). However, the developmental processes and underlying mechanisms between identity exploration and college adaptation over time remain empirically underexplored. Traditional cross-lagged models often conflate between-person differences with within-person processes, limiting the ability to infer intra-individual developmental changes (Hamaker et al., 2015). Therefore, this study aims to investigate the within-person longitudinal associations between self-identity exploration and college adaptation, and to test the mediating role of psychological resilience among Chinese college freshmen using a three-wave longitudinal design and a Random Intercept Cross-Lagged Panel Model (RI-CLPM) (Keijsers and van Roekel, 2018).

### 1.1 Associations between self-identity exploration and college adaptation

Self-identity exploration is the active process by which individuals examine different values, beliefs, and life possibilities before forming a stable sense of self (Arnett, 2014; Marcia, 1966). Constructive forms of exploration, such as exploration in breadth and depth (Luyckx et al., 2008), reflect active, goal-directed engagement with identity alternatives. During the transition to college, engaging in constructive identity exploration helps students clarify personal values and life goals.

However, emerging adulthood theory also emphasizes that this developmental period is inherently marked by instability and uncertainty. While identity exploration is theoretically beneficial in the long term, the process itself requires cognitive effort and often entails navigating ambiguity and setbacks (Plate et al., 2023). The relationship between identity exploration and adaptation is therefore unlikely to be straightforward. Exploration involves confronting uncertainty, testing new social roles, and potentially experiencing rejection or failure (e.g., being excluded from a desired peer group or discovering a mismatch between expectations and reality). These experiences, while developmentally normative, may not translate directly into improved adaptation without the presence of adequate psychological resources to process and integrate them. For Chinese college freshmen, who are abruptly transitioning from a highly structured, exam-oriented system (Gaokao) to a self-directed university environment, this sudden demand for self-exploration can be particularly overwhelming (Liu, 2022). Therefore, constructive identity exploration may not necessarily translate directly or immediately into better college adaptation. Instead, its adaptive value may depend heavily on the subsequent developmental mechanisms it activates, highlighting the need to investigate bridging psychological resources (Janowicz et al., 2025).

### 1.2 The mediating role of psychological resilience

To unpack the mechanisms linking identity exploration and college adaptation, we propose that psychological resilience serves as a crucial mediating bridge. Psychological resilience refers to an individual's capacity to adapt, recover, and maintain psychological well-being when encountering adversity or significant stress (Connor and Davidson, 2003; Luthar et al., 2000). Rather than reflecting an innate, static trait, contemporary developmental perspectives view resilience as a dynamic process that can be fostered through active engagement with life challenges (Wu et al., 2013).

We acknowledge that an alternative theoretical perspective would position resilience as a moderator rather than a mediator. In the classical protective-factor framework (Luthar et al., 2000), resilience buffers the negative impact of adversity on developmental outcomes. Under this view, exploration would lead to good adaptation, but only when students possess sufficient resilience to cope with the setbacks that exploration may bring. However, we propose a theoretically distinct developmental resource-accumulation model. In our framework, the key theoretical assumption is that exploration does not have a direct effect on adaptation that is conditionally moderated by resilience. Rather, we posit that constructive exploration serves as a developmental catalyst that actively builds resilience over time, and that resilience subsequently functions as the proximal mechanism associated with better adaptation. This sequential process differs from the buffering model in a critical way: it does not assume that exploration has an inherent direct adaptive effect that resilience merely amplifies or protects. Instead, it proposes that exploration's adaptive value is realized indirectly through the psychological resources it cultivates.

From a developmental perspective, constructive identity exploration encourages students to actively step out of their comfort zones, confront uncertainties, and learn from trial-and-error experiences. This goal-directed engagement may act as a catalyst for building psychological capital, effectively serving as a “stress inoculation” process that may foster resilience (Masten et al., 2021). In turn, when students develop higher levels of psychological resilience, they may be equipped with stronger emotional regulation, positive cognitive appraisals, and effective problem-solving skills (Yu and Liu, 2025). They may become better positioned to interpret transition-related stress as opportunities for growth rather than threats (Serdiuk et al., 2024), potentially contributing to better adaptation over time (Cao et al., 2024; Jacobs and Keegan, 2022). Thus, we posit that constructive identity exploration may exert its adaptive benefits primarily by cultivating psychological resilience, which subsequently may serve as the core mechanism associated with better college adaptation over time.

### 1.3 Overview of the current study

Previous studies have recognized the importance of identity exploration in college adaptation but rarely investigated the longitudinal mediation mechanisms at the within-person level. To fill these methodological and theoretical gaps, the present study followed 665 Chinese college freshmen from a single university across three waves during the first semester of their freshman year. We utilized a Random Intercept Cross-Lagged Panel Model (RI-CLPM) to investigate the within-person longitudinal associations between self-identity exploration, psychological resilience, and college adaptation, meticulously disentangling stable between-person traits (controlling for gender, neuroticism, and extraversion) from within-person developmental processes (Keijsers and van Roekel, 2018).

Based on the theoretical framework and the conceptualization of resilience as a developmental outcome of active exploration, we formulated the following hypotheses:

Hypothesis 1: At the within-person level, self-identity exploration positively predicts subsequent psychological resilience over time.

Hypothesis 2: At the within-person level, psychological resilience positively predicts subsequent college adaptation over time.

Hypothesis 3: At the within-person level, the indirect pathway from self-identity exploration to college adaptation through psychological resilience is significant. We expect that initial exploration is associated with subsequent resilience, which in turn is associated with better adaptation. Given that both H1 and H2 must be supported for this indirect pathway to emerge, H3 serves primarily to quantify the magnitude and precision of the indirect effect rather than to test a logically independent hypothesis.

## 2 Methods

### 2.1 Research design and participants

A three-wave longitudinal design was employed to examine the within-person associations among identity exploration, psychological resilience, and college adaptation. This study was approved by the Ethics Committee of Guangdong Pharmaceutical University (Approval No. 2025KT-65). All procedures were performed in accordance with the ethical standards laid down in the 1964 Declaration of Helsinki and its later amendments. Participants were first-year undergraduate students recruited from a comprehensive key university located in south China. With the assistance of class counselors, an online survey link was distributed to freshmen in September 2025 (Wave 1; 1 month after enrollment), November 2025 (Wave 2), and January 2026 (Wave 3). The approximately two-month interval between waves was selected to align with theoretically meaningful developmental periods during the freshman transition: Wave 1 captured the initial adjustment phase, Wave 2 the mid-semester consolidation period, and Wave 3 the end-of-semester evaluation period. This interval is consistent with prior longitudinal studies on identity development and college adaptation that have demonstrated meaningful within-person changes over similar timeframes (Klimstra et al., 2010; Luyckx et al., 2008). Data collection was completed in January 2026, and all analyses were conducted thereafter. The invitation explicitly explained the study's purpose, the voluntary nature of participation, assurances of confidentiality, and the estimated completion time. Written online informed consent was obtained from all participants prior to the survey.

An a priori statistical power analysis for structural equation modeling (SEM) indicated that a minimum sample size of 350 was required to detect a small-to-medium effect size (RMSEA = 0.05) with 80% power at an alpha level of 0.05. Anticipating potential longitudinal attrition, we oversampled during the initial recruitment. A total of 842 students completed the Wave 1 survey. Participants were excluded if they failed designated attention check items (*n* = 67), completed the survey in an implausibly short time (*n* = 48), or exhibited straight-lining response patterns (*n* = 32). An additional 30 participants were excluded due to incomplete responses across the three waves. After applying these exclusion criteria, 665 participants who provided valid responses at all three waves were retained for the final analysis (retention rate: 79.0%). To assess potential attrition bias, we compared the 665 retained participants with the 177 excluded participants on Wave 1 demographic and key study variables. No significant differences were found in gender distribution (χ^2^ = 1.24, *p* = 0.265), age (*t* = 0.87, *p* = 0.384), or Wave 1 levels of identity exploration (*t* = 1.12, *p* = 0.263), psychological resilience (*t* = 0.76, *p* = 0.448), or college adaptation (*t* = 0.94, *p* = 0.347), suggesting that attrition was not systematically related to the study variables. We confirm that all analyses were conducted both with and without the excluded outliers, and the core findings remained consistent.

The final sample (*N* = 665) consisted of emerging adults with a mean age of 19.01 years (SD = 0.81). Among them, 327 were male (49.2%) and 338 were female (50.8%). Regarding academic disciplines, 32.8% were enrolled in science and engineering, 34.3% in the humanities and social sciences, and 32.9% in medical-related fields. The hypotheses and analytic approach were specified prior to data analysis based on the theoretical framework outlined in the Introduction. While no formal preregistration was filed, all hypotheses were theory-driven and confirmatory in nature.

### 2.2 Measures

We confirm that all measures and questions relevant to the research questions are comprehensively described below. We followed the standard scoring procedures for all established measures without any deviations.

### 2.3 Self-identity exploration

Self-identity exploration was assessed using the exploration subscale from the Dimensions of Identity Development Scale (DIDS) developed by Luyckx et al. (2008). This 10-item subscale primarily captures constructive identity exploration (i.e., exploration in breadth and depth) and is rated on a 5-point Likert scale (1 = not at all true, 5 = completely true). Higher scores reflect greater constructive identity exploration. A sample item is “I think actively about different directions I might take in my life” (highest factor loading: λ = 0.82). The scale has been well-validated in Chinese emerging adult samples (Qiu et al., 2025). In the present study, the scale demonstrated excellent internal consistency across all three waves (Cronbach's αs ranging from 0.89 to 0.91).

### 2.4 Psychological resilience

Psychological resilience was measured using 20 items adapted from the Chinese version of the Connor–Davidson Resilience Scale (CD-RISC; Connor and Davidson, 2003; Yu and Zhang, 2007). Items were rated on a 5-point Likert scale (1 = not true at all, 5 = true nearly all the time), with higher scores indicating higher levels of psychological resilience. A sample item is “I am able to adapt to change” (highest factor loading: λ = 0.74). The reliability and validity of the CD-RISC have been strongly supported in Chinese college student populations (Chen et al., 2013). The internal consistency for this 20-item version in the current study was excellent across all waves (Cronbach's αs = 0.93 to 0.94).

### 2.5 College adaptation

College adaptation was assessed using a subset of items derived from the Chinese College Student Adjustment Scale (CCSAS; Fang et al., 2005). The original scale consists of 60 items covering multiple dimensions of college adjustment. Given that the present study focused on first-year college students, the employment adjustment dimension (9 items) was excluded. The remaining 51 items assessed six dimensions: interpersonal adjustment (10 items), academic adjustment (11 items), campus adjustment (8 items), emotional adjustment (9 items), self-adjustment (8 items), and satisfaction (5 items). Participants responded on a 5-point Likert scale ranging from 1 (disagree) to 5 (agree). In line with the scale's standard scoring protocol (Fang et al., 2005), in which both subscale and total adaptation scores are computed and interpreted against established norms, negatively worded items were reverse-coded and an overall composite score was calculated, with higher scores indicating better college adaptation. A sample item is “I feel comfortable in the university environment” (λ = 0.74). Because the CCSAS was designed and validated to yield an overall adaptation index, the total composite was retained for the primary analyses. In addition, identity exploration correlated uniformly weakly and positively with all six subscales (rs ranging from.099 to.143), with no subscale showing a qualitatively different pattern, and resilience predicted all subscales with comparable magnitude longitudinally (rs = 0.340 to.397). Each subscale also demonstrated acceptable internal consistency (αs ranging from.75 to.86 across waves). As expected given the number of items, the 51-item composite showed high internal consistency across all three waves (Cronbach's αs = 0.96).

### 2.6 Covariates

Based on prior empirical evidence linking personality traits and demographic factors to college adaptation (Lounsbury et al., 2005), we included gender, Neuroticism, and Extraversion as time-invariant covariates. Neuroticism (8 items; e.g., “I get nervous easily”) and Extraversion (8 items; e.g., “I am someone who is talkative”) were measured at Wave 1 using the corresponding subscales from the Big Five Inventory (BFI; John et al., 1991), which has robust psychometric properties in Chinese contexts (Wang et al., 2011). Both subscales demonstrated high reliability (α Neuroticism = 0.89, α Extraversion = 0.88).

### 2.7 Data analysis strategy

Data analyses were conducted using R software (version 4.2) with the lavaan package. First, descriptive statistics and zero-order correlations were computed. Second, longitudinal measurement invariance (configural, metric, and scalar) was assessed using item parcels across the three waves to confirm that the constructs were measured equivalently over time. Item parcels (three per construct) were used rather than individual items to improve the indicator-to-sample-size ratio and reduce model complexity while maintaining adequate representation of each construct's content domain (Little et al., 2002).

Third, a Random Intercept Cross-Lagged Panel Model (RI-CLPM; Hamaker et al., 2015) was employed to disentangle within-person developmental processes from stable between-person differences. Random intercepts were specified for identity exploration, resilience, and college adaptation to capture trait-like, between-person variance. The within-person components represent time-point-specific deviations from each individual's expected score, and the cross-lagged paths among these components estimate within-person temporal associations. Gender, neuroticism, and extraversion were included as covariates on the random intercepts.

Finally, to quantify the longitudinal indirect pathway (Hypothesis 3), we utilized a bootstrapping approach (5,000 resamples) to calculate the bias-corrected 95% confidence intervals for the within-person indirect effect (Wave 1 Exploration → Wave 2 Resilience → Wave 3 College Adaptation). Model fit was evaluated using standard criteria: CFI ≥0.90, TLI ≥0.90, RMSEA ≤ 0.08, and SRMR ≤ 0.08. Standardized effect sizes (β) are reported for all statistical tests to fulfill disclosure requirements.

## 3 Results

### 3.1 Descriptive statistics and correlation analysis

Table 1 presents the descriptive statistics (means and standard deviations) and zero-order Pearson correlations for the variables across the three waves. Self-identity exploration, psychological resilience, and college adaptation showed positive autocorrelations across the time points. Furthermore, self-identity exploration and psychological resilience generally exhibited positive correlations with college adaptation.

**Table 1:** Descriptive statistics and zero-order correlations among key variables.

Variables	M	SD	1	2	3	4	5	6	7	8	9
W1 exploration	3.52	0.73	1								
W2 exploration	3.57	0.71	0.399^***^	1							
W3 exploration	3.50	0.73	0.292^***^	0.344^***^	1						
W1 resilience	3.56	0.69	0.418^***^	0.217^***^	0.108^**^	1					
W2 resilience	3.56	0.67	0.380^***^	0.391^***^	0.169^***^	0.565^***^	1				
W3 resilience	3.58	0.66	0.244^***^	0.387^***^	0.367^***^	0.353^***^	0.514^***^	1			
W1 adaptation	2.67	0.61	0.131^***^	0.184^***^	0.037	0.177^***^	0.181^***^	0.139^***^	1		
W2 adaptation	2.63	0.60	0.032	0.092^*^	−0.087^*^	0.029	0.109^**^	0.099^*^	0.477^***^	1	
W3 adaptation	2.66	0.60	0.155^***^	0.123^**^	0.013	0.179^***^	0.396^***^	0.195^***^	0.330^***^	0.456^***^	1

W1, Wave 1; W2, Wave 2; W3, Wave 3; Higher adaptation scores indicate better college adaptation.

* *p* < 0.05, ^**^p < 0.01, ^***^p < 0.001.

### 3.2 Measurement model and overall model fit

The model demonstrated good fit to the data: χ^2^(21) = 25.80, *p* = 0.214, CFI = 0.997, TLI = 0.991, RMSEA = 0.019, SRMR = 0.018. Standardized item loadings were satisfactory across all constructs and waves (see Table 2). Longitudinal measurement invariance was supported at the scalar level (ΔCFI < 0.010 for each step; see Supplementary Table 1). We tested whether the cross-lagged parameters were stationary across the two time intervals; the overall chi-square difference was significant (Δχ^2^ = 115.15, Δdf = 9, *p* < 0.001), indicating that some paths varied across waves. We therefore report the freely estimated model.

**Table 2:** Standardized item loadings for all constructs (Wave 1).

Construct/subscale	Item	λ
Identity exploration	1	0.695
	2	0.713
	3	0.705
	4	0.756
	…	
Psychological resilience	1	0.706
	2	0.715
	3	0.744
	4	0.629
	….	
College adaptation—interpersonal	9	0.596
	10	0.555
	14	0.606
	15	0.579
	…	
— Academic	4	0.526
	7	0.633
	19	0.617
	23	0.513
	…	
— Campus	13	0.743
	17	0.613
	21	0.577
	27	0.549
	…	
— Emotional	1	0.633
	3	0.601
	11	0.614
	16	0.645
	….	
— Self	5	0.597
	8	0.590
	12	0.669
	18	0.591
	….	
— Satisfaction	2	0.725
	6	0.661
	26	0.618
	41	0.534
	…	

Standardized loadings from item-level confirmatory factor analyses; all loadings *p* < 0.001. Loadings shown are for Wave 1 and were comparable across waves.

Path-level tests indicated that seven of nine paths met stationarity (ps >0.05); see Supplementary Table 2. Two paths showed significant cross-wave variation: the association of resilience with subsequent adaptation strengthened over time, and the association of adaptation with subsequent exploration reversed direction across waves.

Asymmetric effects were examined via Wald tests comparing paired cross-lagged paths (see Supplementary Table 3). Exploration predicted resilience more strongly than the reverse (E → R vs. R → E: Wald χ^2^ = 7.48, *p* = 0.006), and resilience predicted adaptation more strongly than the reverse (R → A vs. A → R: Wald χ^2^ = 8.71, *p* = 0.003). These directional asymmetries are consistent with the proposed mediation sequence.

### 3.3 Between-person associations

In the RI-CLPM framework, random intercepts capture the time-invariant, trait-like between-person differences. At the between-person level, the associations among the random intercepts of self-identity exploration, psychological resilience, and college adaptation were not statistically significant after controlling for the covariates (RI_E–RI_R: r = 0.147, *p* = 0.619; RI_E–RI_A: r = 0.017, *p* = 0.951; RI_R–RI_A: r = −0.176, *p* = 0.731).

### 3.4 Within-person cross-lagged effects

**Figure 1:**
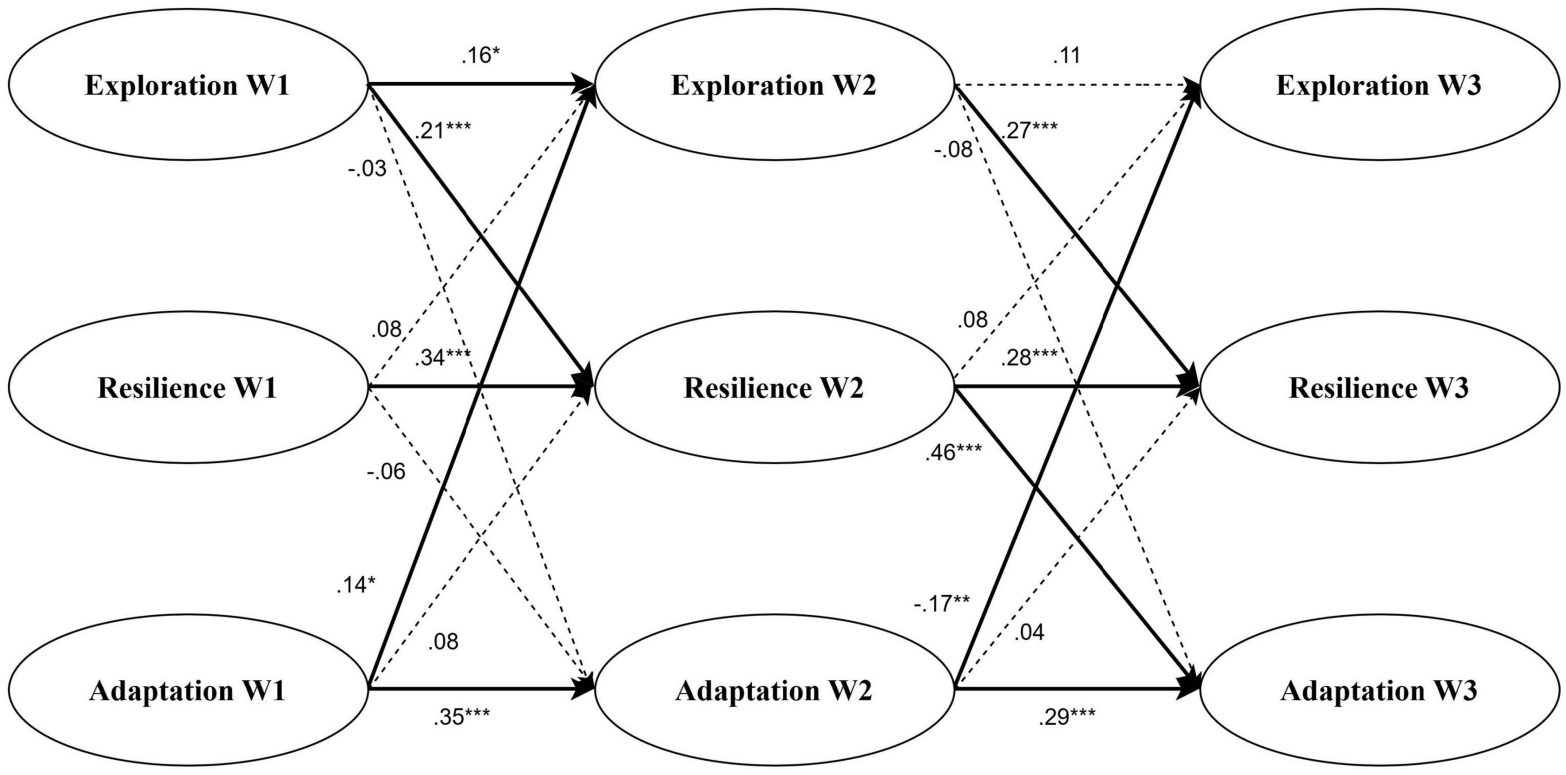
Path diagram of the within-person longitudinal mediation model. This figure presents the within-person structural cross-lagged paths of the RI-CLPM. Solid arrows indicate statistically significant cross-lagged associations (*p* < 0.05), while dashed arrows indicate non-significant paths. The bolded path highlights the focal mediation sequence from Wave 1 exploration to Wave 3 college adaptation via Wave 2 psychological resilience. For visual clarity, the between-person random intercepts, within-person autoregressive paths, within-wave covariances, and covariates are omitted from this graphical representation, although they were fully estimated in the final statistical model. Standardized estimates are presented. *p < 0.05, **p < 0.01, ***p < 0.001.

The within-person cross-lagged paths are presented in Figure 1. Controlling for autoregressive effects, within-person self-identity exploration at Wave 1 showed a positive cross-lagged association with psychological resilience at Wave 2 [β = 0.217, est = 0.203, SE = 0.049, *p* < 0.001, 95% CI (0.105, 0.297)]. This association remained consistent and strengthened from Wave 2 to Wave 3 [β = 0.270, est = 0.260, SE = 0.050, *p* < 0.001, 95% CI (0.163, 0.361)], supporting Hypothesis 1.

Within-person psychological resilience at Wave 2 was positively associated with college adaptation at Wave 3 [β = 0.464, est = 0.420, SE = 0.053, *p* < 0.001, 95% CI (0.320, 0.525)]. However, this association was not significant from Wave 1 to Wave 2 [β = −0.060, est = −0.053, SE = 0.058, *p* = 0.366, 95% CI (−0.169, 0.058)], indicating that the predictive role of resilience for adaptation emerged and strengthened over the course of the semester. Hypothesis 2 was thus partially supported.

The direct cross-lagged associations from self-identity exploration to college adaptation were not statistically significant from Wave 1 to Wave 2 [est = −0.029, *p* = 0.592, 95% CI (−0.134, 0.074)] nor from Wave 2 to Wave 3 [est = −0.074, *p* = 0.083, 95% CI (−0.156, 0.014)].

Among other cross-lagged paths, college adaptation at Wave 1 was positively associated with exploration at Wave 2 (β = 0.141, est = 0.162, SE = 0.069, *p* = 0.019), suggesting that students who adapted well early on had the security to engage in further exploration. Conversely, adaptation at Wave 2 was negatively associated with exploration at Wave 3 (β = −0.176, est = −0.208, SE = 0.075, *p* = 0.006), suggesting that once students consolidated their adjustment by mid-semester, the impetus for continued exploration diminished. The remaining cross-lagged paths (adaptation → resilience, resilience → exploration) were not statistically significant (ps >0.09).

Regarding autoregressive effects, college adaptation showed strong carry-over effects across both intervals (β = 0.352, *p* < 0.001; β = 0.293, *p* < 0.001), as did psychological resilience (β = 0.342, *p* < 0.001; β = 0.286, *p* < 0.001). Identity exploration showed a significant carry-over only in the first interval (β = 0.162, *p* = 0.014) but not the second (β = 0.118, *p* = 0.065).

### 3.5 Longitudinal indirect pathway

To quantify the longitudinal indirect pathway (Hypothesis 3), we tested all six possible within-person indirect paths among self-identity exploration, psychological resilience, and college adaptation using bootstrapping (5,000 resamples). The results are presented in Table 3.

**Table 3:** Within-person indirect effects (bootstrapping 95% CI).

Indirect path (Wave 1 → Wave 2 → Wave 3)	Est.	SE	95% CI	*p*	Std. β
**Exploration** ** → Resilience** ** → Adaptation**	**0.085**	**0.023**	**(0.044, 0.135)**	**<0.001**	**0.101**
Exploration → Adaptation → Resilience	−0.001	0.004	**(**−0.017, 0.003**)**	0.781	−0.001
Resilience → Exploration → Adaptation	−0.006	0.007	**(**−0.029, 0.002**)**	0.399	−0.007
Resilience → Adaptation → Exploration	0.011	0.014	**(**−0.010, 0.045**)**	0.422	0.011
**Adaptation** ** → Exploration** ** → Resilience**	**0.042**	**0.021**	**(0.009, 0.092)**	**0.041**	**0.038**
Adaptation → Resilience → Exploration	0.008	0.010	**(**−0.003, 0.037**)**	0.403	0.007

Significant indirect paths are highlighted in bold.

The analysis indicated a significant indirect pathway from Wave 1 self-identity exploration to Wave 3 college adaptation via Wave 2 psychological resilience [est = 0.085, SE = 0.023, 95% CI (.044, 0.135), *p* < 0.001]. Given that the direct paths from exploration to subsequent adaptation were not significant, this pattern is consistent with a significant longitudinal indirect pathway at the intra-individual level, suggesting that initial constructive identity exploration is associated with subsequent higher psychological resilience, which in turn is associated with better college adaptation over time. Hypothesis 3 was supported.

Additionally, one other significant indirect pathway was observed: from Wave 1 college adaptation to Wave 3 psychological resilience via Wave 2 exploration [est = 0.042, 95% CI (.009, 0.092), *p* = 0.041]. This suggests a reciprocal developmental process in which better initial adaptation may support subsequent exploration, which in turn is associated with greater resilience.

The structural pathways of these within-person cross-lagged processes are visually summarized in Figure 1. The primary indirect pathway of interest is represented by the solid arrows flowing from Wave 1 exploration to Wave 2 resilience, and subsequently to Wave 3 college adaptation. The non-significant direct paths between exploration and adaptation are depicted with dashed arrows, consistent with the indirect pathway structure at the within-person level. The figure also illustrates other significant cross-lagged associations, including the reciprocal pathway from adaptation through exploration to resilience, providing a comprehensive view of the intra-individual temporal dynamics.

## 4 Discussion

The primary objective of this study was to examine the longitudinal associations among constructive self-identity exploration, psychological resilience, and college adaptation during the transition to university. While previous literature has widely documented the importance of identity development during emerging adulthood (Arnett, 2014; Schwartz et al., 2011), the precise mechanisms and within-person temporal sequences linking exploration to college adaptation have remained largely unexamined. The present study utilized a three-wave longitudinal design and a Random-Intercept Cross-Lagged Panel Model (RI-CLPM) to disentangle trait-like between-person differences from intra-individual developmental processes. By doing so, this study extends previous research by suggesting that identity exploration may not be directly associated with immediate improvements in adaptation. Instead, its adaptive value appears to be realized through a significant longitudinal indirect pathway wherein exploration is associated with psychological resilience, which subsequently is associated with better college adaptation over time.

### 4.1 The association between identity exploration and psychological resilience

Our first major finding indicated that, at the within-person level, constructive self-identity exploration positively predicted subsequent psychological resilience. This finding provides an important extension to the existing literature, which has traditionally viewed psychological resilience either as a stable personality trait or as a byproduct of overcoming severe adversity (Luthar et al., 2000; Aburn et al., 2016). The current results support a more dynamic developmental perspective of resilience during emerging adulthood.

When freshmen actively engage in constructive exploration (such as evaluating different academic interests, social groups, and future career paths), they are essentially participating in a normative process of trial and error. Navigating these identity-related uncertainties requires active cognitive engagement and goal reevaluation (Meca et al., 2023). Our findings are consistent with the possibility that this active engagement may function as a form of psychological practice. By stepping out of their comfort zones to explore identity alternatives, students may gradually accumulate coping skills, cognitive flexibility, and a stronger internal locus of control. Over time, these accumulated resources may coalesce into higher psychological resilience. This insight refines the theoretical understanding of resilience by highlighting that proactive self-directed developmental tasks may potentially serve as antecedents for building psychological resources, even in the absence of severe trauma (Ghasemi et al., 2024).

### 4.2 The association between psychological resilience and college adaptation

Consistent with our theoretical expectations, within-person increases in psychological resilience were associated with subsequent better college adaptation, although our freely estimated model revealed that this association demonstrated significant cross-wave variation. While the role of resilience in adaptation is well documented in cross-sectional studies (Connor and Davidson, 2003; Yu and Liu, 2025), our RI-CLPM approach indicated that the association between resilience and subsequent adaptation was not statistically significant during the initial transition (from Wave 1 to Wave 2, b = −0.053, ns), but emerged as a strong positive association over time (from Wave 2 to Wave 3, b = 0.420, *p* < 0.001). One possible interpretation of this delayed pattern is that it reflects a stress inoculation process, wherein newly activated resilience resources require time and continued engagement with the university environment before they become fully associated with better adaptation. However, we note that this interpretation remains speculative, as the present study did not directly measure stress appraisal or coping processes. Alternative explanations, such as the accumulation of environmental stressors over the semester making resilience increasingly relevant, cannot be ruled out. The transition to university presents numerous demands, including academic pressure, novel social environments, and the need for independent living (Ball et al., 2024). Students who experience an increase in their psychological resilience may tend to be better equipped to employ adaptive emotion regulation strategies and reframe stressful situations as manageable demands rather than overwhelming threats (Brites et al., 2024). Consequently, this enhanced internal resource capacity may eventually contribute to helping prevent initial transitional stress from cascading into severe academic, social, or emotional maladjustment later in the academic year.

### 4.3 The indirect pathway through psychological resilience

A notable contribution of this study is the identification of a significant longitudinal indirect pathway. We found that the within-person direct cross-lagged paths from self-identity exploration to college adaptation were not statistically significant; rather, the association was indirectly linked through psychological resilience (Wave 1 Exploration → Wave 2 Resilience → Wave 3 Adaptation, est = 0.085, *p* < 0.001). This finding offers a plausible explanation for the mixed results observed in previous cross-sectional literature, where identity exploration was sometimes linked to positive adaptation and other times associated with increased distress (Plate et al., 2023).

To empirically distinguish between the mediation and moderation frameworks, we conducted supplementary analyses testing the Exploration × Resilience interaction effect on subsequent college adaptation. Using both hierarchical regression and the RI-CLPM framework, the interaction terms were non-significant across both time intervals (all ps >0.36, largest |β| = 0.031; see Supplementary Table 4). These results indicate that resilience does not function as a buffer that conditionally moderates the exploration–adaptation link. Instead, the data are consistent with a sequential resource-accumulation process in which exploration is associated with resilience, which is then associated with better adaptation.

### 4.4 Reciprocal developmental dynamics

Our analysis also revealed a significant indirect pathway from initial college adaptation to later psychological resilience via exploration (Wave 1 Adaptation → Wave 2 Exploration → Wave 3 Resilience, est = 0.042, *p* = 0.041). This finding highlights a more complex, bidirectional developmental process than a simple linear mediation chain. Better initial adaptation at Wave 1 was associated with increased exploration at Wave 2 (b = 0.162, *p* = 0.019), suggesting that a secure early adjustment may give students the stability and confidence to engage in identity exploration. However, better adaptation at Wave 2 was associated with reduced exploration at Wave 3 (b = −0.208, *p* = 0.006), suggesting that once students consolidated a stable adjustment by mid-semester, the impetus for further exploration diminished.

This reciprocal pattern is consistent with developmental systems perspectives that emphasize bidirectional influences among psychological processes over time (Lerner, 2007). The freshman transition may thus involve a dynamic cycle: adaptation and exploration mutually influence each other across different phases of the transition, with resilience serving as a key resource that is both built through exploration and contributes to subsequent adaptation. These findings suggest that freshman adaptation is better understood as a dynamic, reciprocal developmental process rather than a simple unidirectional mediation pathway.

Our results suggest that constructive identity exploration is a demanding developmental task that requires sustained cognitive and emotional effort. For university freshmen who transition from structured secondary education systems into more autonomous university environments, such as those in China and many East Asian countries, the sudden onset of identity exploration may temporarily elevate uncertainty (Liu, 2022). Therefore, simply exploring identity alternatives may not automatically or immediately be associated with better adaptation. Instead, the potential adaptive value of identity exploration appears to be indirect and temporally delayed. Exploration may serve as the necessary groundwork for cultivating psychological resilience. Once this resilience is developed, it may act as the proximal mechanism associated with better college adaptation (Hanimoglu, 2025). This indirect pathway model shifts the theoretical focus from expecting direct outcomes of exploration to understanding the essential intervening psychological resources that may be required for successful adaptation.

### 4.5 The dynamics of autoregressive patterns

A noteworthy pattern emerged in the autoregressive paths that refines our understanding of these developmental constructs. Both psychological resilience and college adaptation exhibited significant and stable autoregressive effects across the intervals. College adaptation demonstrated strong carry-over effects (β = 0.352 and.293, ps < 0.001), indicating that without intervention, early adaptation patterns tend to persist. Psychological resilience similarly showed robust self-sustaining properties (β = 0.342 and.286, ps < 0.001), where gains in resilience tend to be retained rather than dissipating. Meanwhile, identity exploration showed a significant carry-over only in the first interval (β = 0.162, *p* = 0.014) but not the second (β = 0.118, *p* = 0.065).

This pattern carries implications for the indirect pathway mechanism. The strong autoregressive stability of adaptation highlights the tendency for students to maintain their adaptation patterns over time. However, the E → R → A indirect pathway demonstrates how change may occur: identity exploration is associated with elevated resilience at Wave 2; resilience then utilizes its own self-sustaining momentum and shows a strong positive association with subsequent adaptation at Wave 3 (β = 0.464). The fact that resilience is significantly associated with subsequent adaptation even when controlling for the strong autoregressive carry-over of prior adaptation underscores the potential importance of resilience as a dynamic, accumulable resource that may bridge the exploration process with adaptive outcomes over time.

### 4.6 Broader implications across educational contexts

Although the present study was conducted with Chinese university freshmen, the core findings may offer relevant insights for higher education institutions in other countries and regions. The transition from secondary to tertiary education universally requires students to navigate increased autonomy, forge new social relationships, and reconsider personal goals and values (Arnett, 2014; Credé and Niehorster, 2012). These identity-related demands are not unique to the Chinese educational context. In particular, students in other East Asian systems, such as those in South Korea and Japan, undergo a similarly abrupt shift from highly structured, examination-driven secondary schooling to relatively autonomous university environments, making the exploration-resilience-adaptation pathway identified here potentially applicable. Moreover, even in Western educational settings where student autonomy is emphasized earlier, emerging adults still face identity exploration as a central developmental task during the college years (Schwartz et al., 2015). The indirect pathway we observed, in which exploration is associated with adaptation not directly but through resilience building, may therefore represent a potentially generalizable process. However, given that the present sample was drawn from a single university in southern China, these cross-contextual implications remain tentative. Future research should replicate this model across diverse cultural and institutional contexts to determine whether the timing and magnitude of these within-person effects vary as a function of educational structure, cultural values, or institutional support systems.

### 4.7 Practical implications

The findings of this study have tentative practical implications for higher education institutions broadly, with particular relevance to Chinese colleges, regarding how to more effectively support freshmen adaptation. To support college freshmen in becoming active members of their college communities and to facilitate a smooth transition into college life, colleges typically offer various new student orientation activities before or at the beginning of the first semester (Barefoot, 2005). In China, colleges‘ new student orientation activities usually include various lectures that introduce the colleges' history, service resources, campus safety actions, mental health promotion initiatives, and ethical requirements. However, our study suggests that supporting self-identity exploration at the beginning of college may be relevant, as it may potentially help build freshmen's psychological resilience and contribute to better adaptation over time.

Based on our findings, we tentatively suggest that orientation activities at universities, including but not limited to those in China, could consider placing greater emphasis on teaching goal-setting skills and fostering self-reflection habits that are essential for university success but may not be emphasized in high school environments. For example, it may be advisable for colleges to set up workshops or seminars on collaborative learning, academic planning, and values clarification to equip freshmen with the skills and strategies necessary for successful adaptation (Aquino, 2011). These types of structured exploration programs are not context-specific and could be adapted to universities in other regions where freshmen similarly face the challenge of transitioning from externally regulated learning to self-directed academic life (Tinto, 2012; Bowman, 2010). In addition to providing direct exploratory guidance, it may be advisable for colleges to implement strategies to help freshmen develop character strengths, particularly a sense of meaning in life and psychological resilience, during their early college experience. A stronger sense of meaning can motivate students to autonomously engage in university life and may contribute to their overall adaptation (Ti et al., 2025).

Furthermore, our study revealed that the association between psychological resilience and college adaptation was not immediate but appeared to emerge and strengthen over the course of the freshman year (R → A: *b* = −0.053, ns at T1 → T2; *b* = 0.420, *p* < 0.001 at T2 → T3). This delayed pattern carries a potentially relevant practical message: a single mental health screening administered shortly after enrollment may be insufficient, as resilience differences may not yet be associated with observable adaptation differences at that early stage. Instead, universities, whether in China or elsewhere, could consider implementing repeated mental health and resilience assessments across the freshman year, ideally at least once per semester, to capture the point at which low resilience begins to be associated with tangible difficulties. Such a longitudinal monitoring system could potentially enable timely identification of at-risk students and allow institutions to provide targeted professional support before maladjustment becomes severe, rather than responding only after problems have fully emerged. Although the timing of this association may vary across educational and cultural contexts, the general principle of longitudinal monitoring over single-point screening may be broadly applicable to higher education institutions worldwide.

### 4.8 Limitations and future research

Several limitations of the current study should be noted, providing important directions for future research. First, although the RI-CLPM strengthens the longitudinal analysis by separating within-person from between-person effects, the study remains observational and cannot establish causality. The findings represent longitudinal within-person associations rather than causal effects. Experimental or intervention designs would be needed to establish causal claims.

Second, the sample was drawn from a single comprehensive university in southern China. Although the sample size was adequate and the longitudinal design was robust, the unique institutional context may limit the generalizability of the findings. The transition experience for students in vocational colleges or universities in different cultural contexts may exhibit different developmental patterns based on varying educational expectations (Arnett, 2014). Future research should replicate this longitudinal model across diverse institutional types and cultural backgrounds to verify the generalizability of the indirect pathway.

Third, the study retained 665 matched responses from 842 initial participants (79.0% retention rate). Although comparisons between retained and excluded participants on baseline characteristics revealed no significant differences, we cannot entirely rule out the possibility that selective attrition may have influenced the findings in ways not captured by the baseline variables examined.

Fourth, the study relied entirely on self-report measures. While established and validated scales were utilized, self-reported data may be subject to social desirability or shared method variance. Additionally, the college adaptation measure was derived from a positive adjustment scale. Future studies would benefit from incorporating multi-informant designs, such as peer nominations, teacher evaluations, or objective academic and disciplinary records, to provide a more comprehensive assessment of college adaptation. Additionally, utilizing daily diary methods or ecological momentary assessment (EMA) could capture the micro-level daily fluctuations in identity exploration and emotional well-being, offering deeper insights into how exploration occurs in real time (Myin-Germeys et al., 2018).

Fifth, the longitudinal timeframe covered a single academic semester during the initial transition to university. While this initial five-month period is a critical window for early adaptation, identity development and university adaptation are protracted processes that continue throughout the entire college experience. Future researchers should consider employing longer-term longitudinal designs spanning multiple years with more measurement waves to capture the broader developmental trajectories of emerging adults. Moreover, while the RI-CLPM effectively separates within-person effects from between-person differences, it assumes that the cross-lagged parameters are invariant across all individuals. Future research could utilize dynamic structural equation modeling (DSEM) to capture individual-specific pathways and account for greater heterogeneity in adaptation trajectories (Asparouhov et al., 2018).

Finally, this study focused exclusively on constructive identity exploration, encompassing exploration in breadth and depth. Identity theory also identifies ruminative exploration, which is characterized by repetitive, anxiety-driven questioning without commitment (Luyckx et al., 2008). Ruminative exploration may exhibit a different longitudinal pattern, potentially depleting psychological resilience and being associated with poorer adaptation. Future research should compare constructive and ruminative exploration to provide a more nuanced understanding of the multifaceted nature of identity development during emerging adulthood.

## 5 Conclusion

This study advances the understanding of college freshman adaptation by examining the longitudinal, within-person associations linking self-identity exploration to college adaptation. Utilizing a three-wave RI-CLPM, the findings indicate that constructive identity exploration is not directly associated with better adaptation at the within-person level. Instead, it may serve as a developmental antecedent that is associated with psychological resilience, which in turn is positively associated with subsequent college adaptation. The findings also reveal reciprocal dynamics, with adaptation and exploration mutually influencing each other across different phases of the transition. These findings highlight the importance of viewing identity exploration and resilience building as interconnected processes, offering preliminary insights that may inform university support systems aimed at facilitating healthy transitions during emerging adulthood.

## Data Availability

The data presented in this study are available on request from the corresponding author due to privacy and ethical restrictions, as the dataset contains sensitive psychological information of university students. Requests to access the datasets should be directed to Yanting Huang (huangyanting@hncfs.edu.cn).
