# Supplementary material for: How identity exploration may support freshman adaptation: a longitudinal within-person mediation through psychological resilience
Source: Front Psychol. 2026 Jul 8;17:1841811. doi: 10.3389/fpsyg.2026.1841811 (PMC13393315; doi:10.3389/fpsyg.2026.1841811)
Supplement: Supplementary file 1 [file Data_Sheet_1.pdf]

Supplementary Table 1. Longitudinal Measurement Invariance Tests

Model	χ^2	*df*	CFI	TLI	RMSEA	SRMR	ΔCFI	ΔRMSEA	Decision
1. Configural	347	261	.995	.994	.022	.021	—	—	Baseline
2. Metric	357	273	.995	.994	.022	.022	.000	.000	Supported
3. Scalar	378	291	.995	.994	.021	.023	.000	-.001	Supported

Note. Correlated uniquenesses were included for identical parcels across waves. Invariance was evaluated using |ΔCFI| < .010 and ΔRMSEA < .015. Metric vs. configural: $\Delta\chi^2(12) = 11.28$, p = .505. Scalar vs. metric: $\Delta\chi^2(18) = 20.18$, p = .323.

Supplementary Table 2. Stationarity Constraint Tests: Path-Level Wald Tests

Path	T1→T2 *b*	T2→T3 *b*	Wald $\chi^2(1)$	*p*	Stationary?
Autoregressive					
E → E	.157*	.120	0.31	.576	Yes
A →A	.350***	.295***	0.87	.352	Yes
R → R	.337***	.285**	0.83	.364	Yes
Cross-lagged					
E → R	.203***	.260***	0.93	.336	Yes
E →A	-.029	-.074	0.59	.441	Yes

R → E	.083	.090	0.01	.926	Yes
R → A	-.053	.420***	48.74	< .001	No
A → E	.162*	-.208**	23.02	< .001	No
A → R	.089	.041	0.65	.422	Yes
Overall			115.15 (Δ*df* = 9)	< .001	
ΔCFI = −.064			ΔRMSEA = .009		

Note. * p < .05, ** p < .01, *** p < .001. Stationarity is supported when the Wald test is non-significant (p ≥ .05). The overall omnibus test compares the freely estimated model (χ^2 = 25.80, df = 21) with the fully constrained stationary model (χ^2 = 140.95, df = 30).

Supplementary Table 3. Asymmetric Effects: Wald Tests Comparing Paired Cross-Lagged Paths

Variable Pair	Direction A	*b*~A~	Direction B	*b*~B~	Wald $\chi^2(1)$	*p*	Conclusion
E ↔ R (pooled)	E → R	.463	R → E	.173	7.48	.006	E→R stronger
R ↔ A (pooled)	R → A	.367	A → R	.130	8.71	.003	R→A stronger

E ↔ A (pooled)	E →A	-.103	A → E	-.046	0.34	.562	Symmetric

Note. Pooled estimates represent the summed unstandardized coefficients across the two time intervals (T1→T2 + T2→T3). A significant Wald test ($p < .05$) indicates that the two reciprocal paths differ significantly in magnitude. E = Self-identity exploration; R = Psychological resilience; A = College adaptation.

Supplementary Table 4. Moderation Tests: Exploration × Resilience Predicting College Adaptation

**Analysis**	**Interaction b**	**Std. β**	**p**	**Significant**
T1 E×R → T2 Adaptation (hierarchical regression)	.008	—	.796	No
T2 E×R → T3 Adaptation (hierarchical regression)	.027	—	.361	No
T1 E×R → T2 Adaptation (RI-CLPM)	.003	.007	.854	No
T2 E×R → T3 Adaptation (RI-CLPM)	.016	.031	.369	No

Note. Mediation model AIC = 10550.2; moderation model AIC = 10553.4. All interaction terms were non-significant; the largest standardized interaction coefficient was $\beta = .031$. The non-significant interactions, combined with the significant sequential indirect effect, support a resource-accumulation (mediation) process rather than a buffering (moderation) process.
